# Calcium Influx of Mast Cells Is Inhibited by Aptamers Targeting the First Extracellular Domain of Orai1

**DOI:** 10.1371/journal.pone.0158223

**Published:** 2016-07-08

**Authors:** Renshan Sun, Yongqiang Yang, Xinze Ran, Tao Yang

**Affiliations:** 1 Department of Dermatology, Institute of Battle Surgery, Daping Hospital, Third Military Medical University, Chongqing, 400042, China; 2 Institute of Combined Injury, College of Military Preventive Medicine, Third Military Medical University, Chongqing, 400038, China; Cornell University, UNITED STATES

## Abstract

Using the systematic evolution of ligands by exponential enrichment (SELEX) method, we identified oligonucleotides that bind to the first extracellular domain of the Orai1 protein with high affinities and high specificities. These ligands were isolated from a random single-strand DNA (ssDNA) library with 40 randomized sequence positions, using synthesized peptides with amino acid sequences identical to the first extracellular domain of the Orai1 protein as the targets for SELEX selection. Seven aptamers were obtained after 12 rounds of SELEX. An enzyme-linked oligonucleotide assay (ELONA) was performed to determine the affinities of the aptamers. Aptamer Y1 had the highest affinity (Kd = 1.72×10^−8^ mol/L) and was selected for functional experiments in mast cells. Using LAD2 cells with the human high-affinity IgE receptor and Ca^2+^ release activation channel (CRAC), we demonstrated that Aptamer Y1 blocked IgE-mediated β-hexosaminidase release from cells triggered by biotin-IgE and streptavidin. A specific binding assay showed that Aptamer Y1 not only bound the Orai1 peptide specifically but also that the Orai1 peptide did not bind significantly to other random oligonucleotide molecules. Furthermore, Aptamer Y1 regulation of intracellular Ca^2+^ mobilization was investigated by probing intracellular Ca^2+^ with a Fluo-4-AM fluorescent probe. We found that Aptamer Y1 inhibits Ca^2+^ influx into antigen-activated mast cells. These results indicate that the target of Aptamer Y1 in the degranulation pathway is upstream of Ca^2+^ influx. Therefore, these oligonucleotide agents represent a novel class of CRAC inhibitors that may be useful in the fight against allergic diseases.

## Introduction

Mast cells are major effectors in allergic responses. Precise Ca^2+^ signalling and store-operated Ca^2+^ entry (SOCE) are crucial for proper mast cell function [1,2]. The molecular basis underlying SOCE consists of Ca^2+^ sensor proteins (the stromal interaction molecules STIM1 and STIM2) in the endoplasmic reticulum (ER) and the Orai Ca^2+^ channels in the plasma membrane [3,4]. The protein Orai1 was identified in 2006 and confirmed to be a key component of the Ca^2+^ release activation channel (CRAC) [5,6]. Orai1 is a plasma membrane protein with four predicted transmembrane domains and intracellular N- and C-termini. It has been shown that when either of the amino acid codons D110 and D112, which confer a negative charge in the first extracellular loop of Orai1, are mutated to a codon for glycine and then this mutant cDNA is transfected into HEK293 cells, Orai1 loses its calcium-channel function [5–8]. Therefore, Orai1 calcium channel function may be inhibited by chemical compounds that bind the first extracellular domain of the Orai1 protein and thus change the characteristics of the domain’s charge. In the 1990s, aptamers screened by systematic evolution of ligands by exponential enrichment (SELEX) were found to bind target molecules with high specificity, high affinity and without immunogenicity [9,10]. In this study, peptides of the first extracellular domain of Orai1 were utilized as bait to screen aptamers by SELEX, and the effects of the aptamers on the calcium entry and degranulation of mast cells were investigated. This work verifies the potential of aptamers to become a new class of potent therapeutic agents in the fight against mast cell-mediated diseases.

## Materials and Methods

### Reagents

The first extracellular domain of the Orai1 protein, whose sequence is DADHDYPPGLL [8], was synthesized by Parkson Technology Co., Ltd. (Beijing, China), and this Orai1 peptide was used as a target for SELEX selection. Two other short peptides, Cd1215 and IgEop, with sequences of LIPTHTQPSY and GTYYCTGKVWQLDYE, respectively, were synthesized by the same company and were used separately in an aptamer-specific assay. A random ssDNA library and primers were synthesized by the Yingjun Biotechnology Company (Shanghai, China). Three random oligonucleotides, designated ONT1, ONT2 and ONT3, were synthesized by the same company. The sequences of these three oligonucleotides are TCA AGC TTA TGA CCG AGC GCC GCG TGC C, TAG GAT CCC CAG ACC TGC CCG CCA TGT, and GGT ACC GCA GAA TTG GGA AGA GAT AGA, respectively. Biotinylated oligonucleotides were synthesized by Briggs (Shanghai, China). The pGEM-T easy vector was obtained from TaKaRa (Dalian, China). STEMPRO-34 SFM Complete Medium and the Ca^2+^ fluorescent probe Fluo-4-AM were purchased from Invitrogen (Carlsbad, CA, USA). A DNA extraction kit was obtained from Tiangen Biotech Co., Ltd. (Beijing, China). HRP-labelled streptavidin was purchased from Biyuntian (Tianjin, China). Biotin-IgE was purchased from US Biologicals (St. Louis, MO, USA). Human stem cell factor was purchased from Peprotech (Rocky Hill, USA). Streptavidin was obtained from Sangon Biotech Co. Ltd. (Shanghai, China). Polystyrene microwell 96-well detachable ELISA plates were purchased from Corning (Shanghai, China). The SELEX screening buffer liquid and Tyrode's solution were prepared according to the method described by Pan Q et al [11]. The reagents for cell cultures were purchased from Sigma-Aldrich.

### Methods

#### Construction of the single-stranded DNA (ssDNA) library

Single-stranded (ss) DNA library construction was performed as previously described [11]. An oligonucleotide template was synthesized as a single-stranded 81-mer fragment with the sequence 5’-CTTCTGCCCGCCTCCTTCC(40N)-GGAGACGAGATAGGCGGACACT-3', in which the central N40 represents incorporated random oligonucleotides based on equal proportions of A, G, C and T. The PCR amplification primers used to obtain the double-stranded DNA included the forward primer, which was identical to the 5' flanking sequence of the library template (5'-CTTCTGCCCGCCTCCTTCC-3'), and the reverse primer, which was complementary to the 3' flanking sequence of the library template (5'-AGTGTCCGCCTATCTCGTCTCC-3'). The biotin-labelled downstream primer was 5'-biotin-AGTGTCCGCCTATCTCGTCTCC-3’. The double-stranded products were separated into single-stranded DNA fragments via asymmetric PCR.

#### System evolution of ligands by exponential enrichment (SELEX) selection

The selection of DNA aptamers with high affinities against the Orai1 peptide was performed as previously described [9,11]. ELISA plates (96-well) were coated with the Orai1 peptide (in 100 μL of 0.1 mol/L NaHCO_3_ buffer, pH 9.4) by incubating overnight at 4°C. Control wells were coated with BSA. The wells were then rinsed four times each with washing buffer (PBS containing 0.05% Tween 20, pH 7.4; PBST) and incubated for 1 h at room temperature with 200 μL of blocking buffer (PBS containing 3% BSA and 0.05% Tween 20, pH 7.2). The ssDNA pools were denatured by heating at 90°C for 5 min in SHCMK binding buffer (20 mmol/L Hepes, pH 7.35, 120 mmol/L KCl, 1 mmol/L CaCl_2_, and 1 mmol/L MgCl_2_) and then cooled to room temperature for 15 min. The ssDNA library was first added to control wells and incubated at 37°C to screen out ssDNA fragments that targeted BSA. The unbound ssDNA was then removed and placed in the Orai1 peptide-coated wells for incubation at 37°C. Unbound ssDNA sequences were removed by rinsing six times with washing buffer (SHCMK supplemented with 0.05% Tween 20; SHCMKT). Then, the Orai1 peptide-bound ssDNA was recovered by incubating with eluting buffer (20 mmol/L Tris–HCl, 4 mol/L guanidinium isothiocyanate, and 1 mmol/L DTT, pH 8.3) at 80°C for 10 min. The eluates were mixed with phenol-chloroform and centrifuged at 12,000×g for 5 min at 4°C. The resulting supernatants were mixed with dehydrated alcohol and NaAc (3 mol/L, pH 5.2) overnight at −20°C, followed by centrifugation at 12,000×g for 20 min at 4°C. The supernatants were discarded and the pellet was resuspended in 75% alcohol and centrifuged for 10 min. The precipitate was then dissolved in 30 μL of TE buffer (pH 8.0) and applied as a template for PCR amplification with biotin-labelled primers. The amplification thermal cycling parameters were as follows: one cycle of 94°C for 5 min; 18 cycles of 94°C for 30 s, 65°C for 30 s, and 72°C for 30 s; and one cycle of 72°C for 5 min. Asymmetric PCR with unequal molar concentrations of the forward and reverse primers was carried out to separate the ssDNA. The asymmetric thermal cycling parameters were as follows: one cycle of 94°C for 5 min; 35 cycles of 94v for 30 s, 65°C for 30 s, and 72°C for 30 s; and one cycle of 72°C for 5 min. The ssDNA products were used as the enriched library for the next round of selection.

#### Cloning and sequencing

After several rounds of aptamer selection, the PCR products were purified individually from each round and cloned into a pMD 19-T vector using a TA cloning kit. After transformation into E. coli DH5α cells, individual bacterial clones were chosen from each of the aptamer selection rounds for sequencing.

#### Affinity and specificity determinations

To determine the affinities of the aptamers, an enzyme-linked oligonucleotide assay (ELONA) was carried out according to a previous publication [12]. Polystyrene microwell plates were coated with Orai1 peptide (10 μg in 100 μL of 0.1 mol/L NaHCO_3_ buffer, pH 9.4) and saturated with blocking buffer. After six rinses with PBST, the biotin-labelled aptamers (in SHCMK buffer) were added at 0.1 μg/well. The aptamers and the Orai1 peptide were allowed to react for 3 hours at 37°C. The wells were then rinsed six times with SHCMKT buffer, and 100 μL of streptavidin-horseradish peroxidase (HRP; 1:1000 in PBS) was added. The plates were incubated for 40 min at 37°C. After the wells were rinsed six times with PBST, a mixture of 100 μL of 1 mmol/L tetramethylbenzidine (0.1 mol/L in citrate buffer, pH 4.25) and 2 mmol/L H_2_O_2_ (1:20 ratio) was added as substrate. The enzymatic reaction was stopped 10 min later by adding 50 μL of 1 mol/L H_2_SO_4_, and the optical density (OD) at 450 nm was determined with a spectrophotometer.

#### Determination of the dissociation constant (Kd) values of individual ssDNA aptamers [12]

The ssDNA aptamers were amplified with a biotin-labelled primer to generate biotin-labelled ssDNA aptamers. ELISA plates were coated with Orai1 peptide (0.01 mg/mL). Various concentrations of biotin-labelled single aptamers were added and incubated at 37°C for 2 h. HRP-conjugated streptavidin (1:1000) was added and incubated for 30 min at 37°C. After adding substrate and stop buffer, the absorbance was determined at 450 nm using a microplate reader. The apparent Kd values were determined by nonlinear regression for on-site binding according to the equation Y = Bmax * X/(Kd + X), using GraphPad Prism version 5.0 (GraphPad Software, Inc.). Non-specific targets to test specificity included Cd1215, IgEop and BSA. Additional specificity testing was performed using different biotinylated oligonucleotides, specifically ONT1, ONT2 and ONT3. These oligonucleotides were used as probes to bind the Orai1 peptide-coated wells.

#### Effects of nucleic acid aptamers on IgE-mediated β-hexosaminidase release [13,14]

LAD2 cells were provided by Dr. Michael D. Gershon from Columbia University. The cells were cultured in 50-mL culture flasks with 5 mL of complete media in each bottle. The complete media contained Stem Pro-34 Nutrient Supplement, 100 U/mL penicillin, 100 μg/mL streptomycin, 2 mmol/L glutamine, and 100 ng/mL human stem cell factor. The cell density was maintained at 0.5×10^6^/mL, and half of the culture media was removed and replaced with fresh culture media weekly. LAD2 cells were centrifuged at 3000 rpm (Hettich, Rotofix 32, swing-out rotor), resuspended in Tyrode’s buffer at 2 × 10^6^ cells/mL and primed overnight with 500 ng/mL biotinylated human myeloma IgE. Cells were activated the following morning with 500 ng/mL streptavidin and incubated for an appropriate length of time, and the reaction was stopped by centrifuging the cells at 3000 rpm for 3 min. To evaluate the effects of oligonucleotides on mast cell degranulation, the aptamers were dissolved in PBS to the appropriate concentration and added to the cells 2 min prior to cell activation with streptavidin. β-Hexosaminidase release was assessed by measuring samples at 405 nm using a microplate spectrophotometer and expressed as a percentage of the total cellular β-hexosaminidase.

#### Effects of aptamers on calcium influx into LAD2 cells as determined by laser confocal microscopy [13,14]

LAD2 cells were incubated with the Ca^2+^ fluorescent probe Fluo-4-AM at 5 μmol/L for 30 min at room temperature. After washing with Tyrode’s solution three times, the dye inside the cells was allowed to de-esterify for 30 min at 37°C. Fluorescent images of Ca^2+^ were obtained using an Olympus 1000 confocal microscope with a 40× oil immersion lens (NA 1.3) (Olympus, Japan). The Fluo-4-AM signal was excited at 488 nm and emitted at > 505 nm. Frame-scan images were acquired at a sampling rate of 15 ms per frame and 20 s per interval. Image data were analysed off-line using fv10-asw.2.1 software. A selected image from each image set was used as a template to designate the region of interest (ROI) within each cell. The integrated intracellular Ca^2+^ concentration was determined by calculating ΔF/F0. F0 was defined as the mean basal fluorescence intensity of the dye recorded during the first 5–10 scanning frames, when the cells were under rest conditions. ΔF denotes (F-F0), where F is the temporal fluorescence intensity. The ΔF/F0 values within each ROI were plotted as a function of time (typical time course of Ca^2+^ response to streptavidin stimulation in single LAD2 cells). The amplitude of the Ca^2+^ response within each cell was quantified as the highest ΔF/F0 level reached during the measurement period, which was averaged over all cells within each group. The procedure was performed using a previously reported method with modifications [14]. Observation of the cells by microscopy was conducted immediately after activation with 500 ng/mL streptavidin. During the observation process, an image was collected every 5 s. The baseline was collected 1 min before the addition of streptavidin, and the observation period lasted approximately 4 min after the addition of streptavidin.

#### Statistical analysis

SPSS 13.0 statistical software was used for data analysis, and the data are expressed as $\bar{x}$ ± s. Two-tailed Student’s t-tests and analysis of variance (ANOVA) were used for comparisons, and *P*-values of 0.05 or less were regarded as statistically significant.

## Results

### Optimization of the PCR amplification conditions

Optimization of the asymmetric PCR amplification conditions was important for the generation and screening of the ssDNA secondary library (ssDNA was amplified by symmetric PCR to dsDNA, and then the dsDNA was amplified by asymmetric PCR to ssDNA). Visible bands were obtained from PCR reactions performed with annealing temperatures of 53.3–65.3°C; when the annealing temperature reached > 65°C, PCR product quantities began to decrease. We chose 64.3°C as the most appropriate annealing temperature and performed 35 cycles of amplification. Based on the appearance of the indirect asymmetric PCR products in agarose gels, a molar concentration proportion of 1:50 for both the forward and reverse primers was chosen to obtain a maximum number of specific PCR products with the formation of only a minimal number of PCR by-products.

### SELEX screening

**Fig 1:**
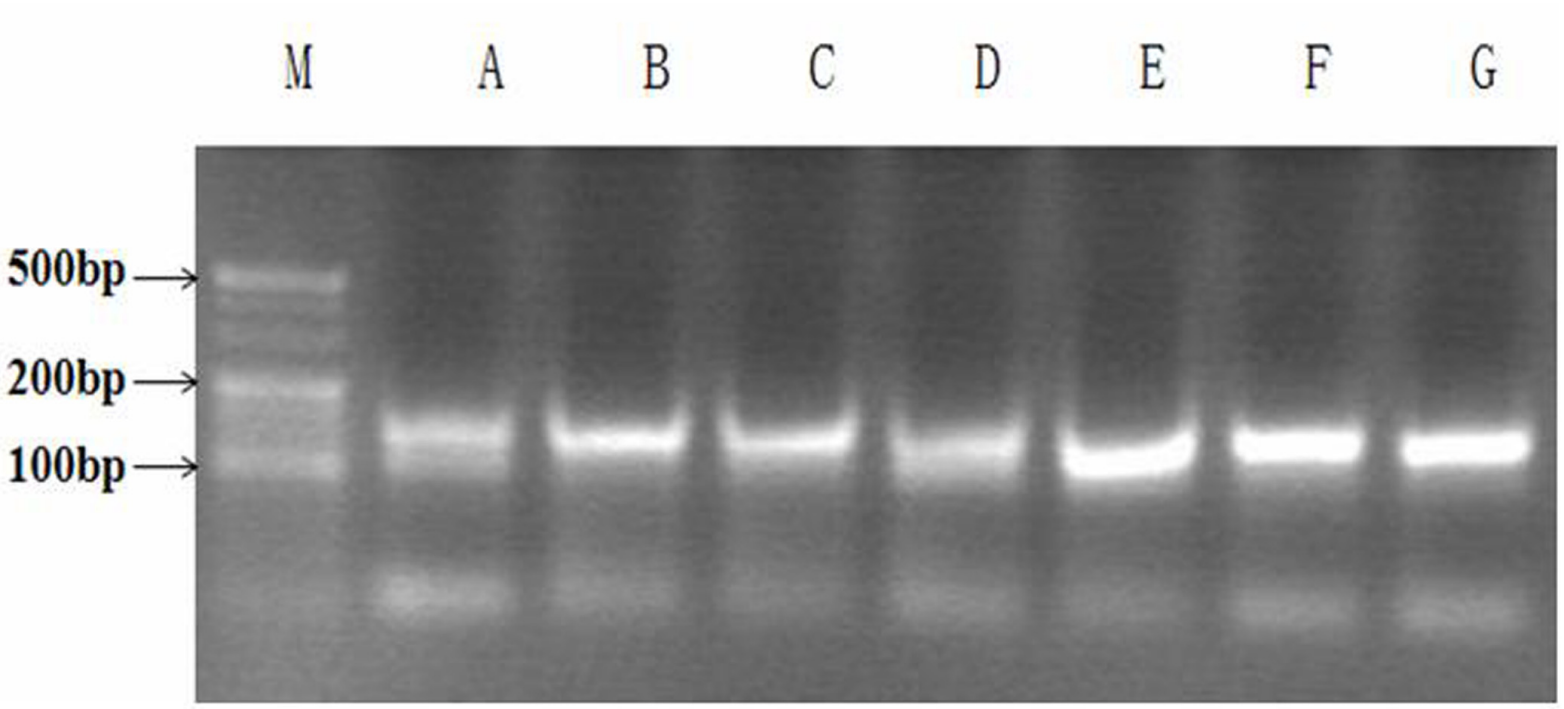
Agarose gel (3%) electrophoretic analysis of polymerase chain reaction (PCR) products of aptamer pools. Lanes A-G: aptamer pool PCR products from the 1st, 3rd, 5th, 7th, 9th, 11th and 12th rounds of SELEX selection; lane M: DNA ladder.

**Fig 2:**
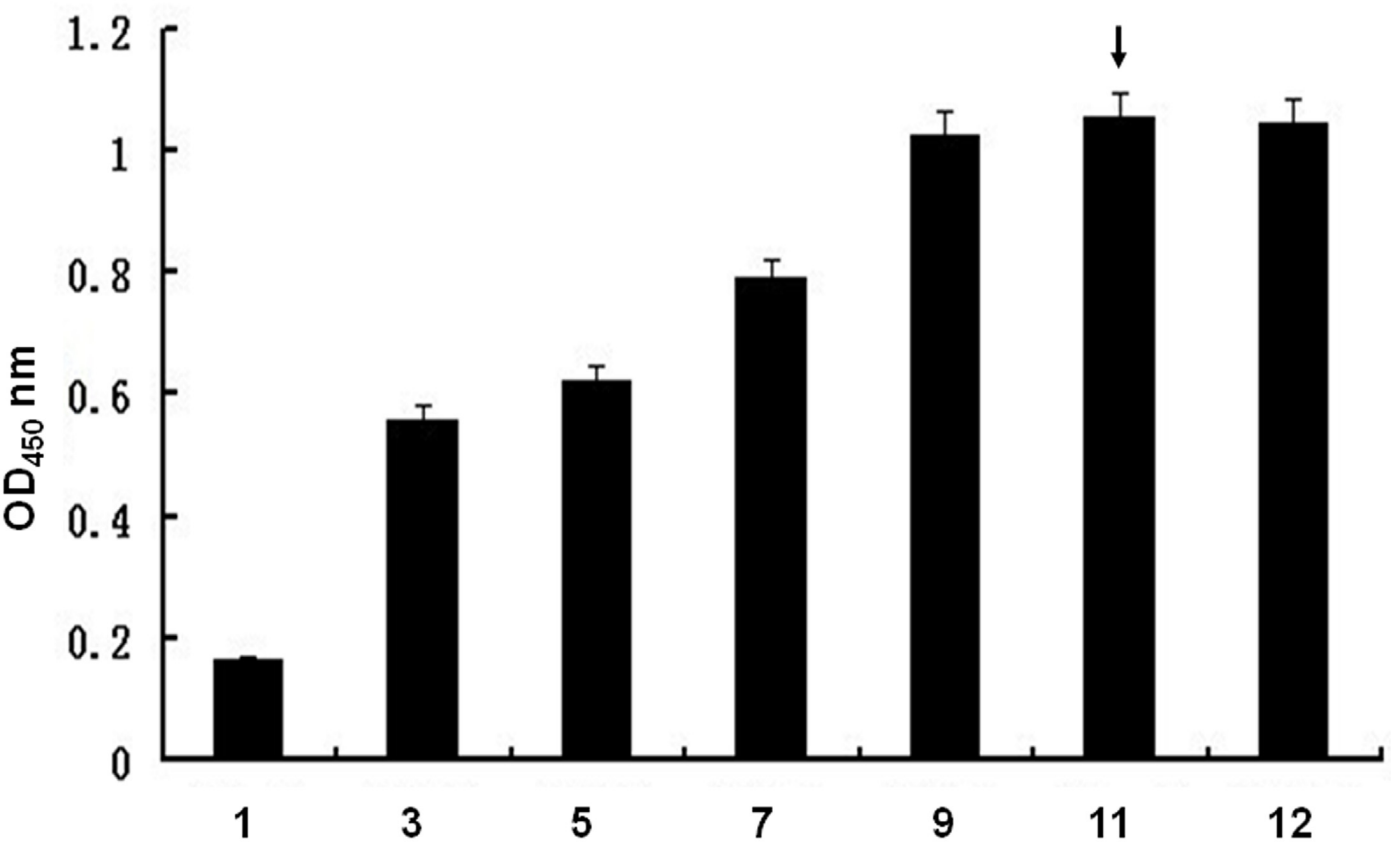
Relative binding affinities of the ssDNA pools to the Orai1 peptide as determined by ELONA analysis. Orai1 peptide was coated onto a 96-well microplate, and biotin-labelled aptamer pools (from rounds 1, 3, 5, 7, 9, 11 and 12) were added into the wells individually. The absorbance was determined at 450 nm. All data shown were calculated as the mean ± SEM, and data were obtained from three independent experiments. The highest relative affinities of the aptamers are indicated by arrows.

To isolate aptamers that specifically bind to the Orai1 protein, we utilized the Orai1 peptide as a selection target. BSA was used as a negative control target. The random ssDNA library was used to screen ligands that bind to the Orai1 peptide. Different pools of aptamers that bind to the Orai1 peptide were obtained through 12 rounds of SELEX selection. Each pool of aptamers was examined on a 3% agarose gel. The aptamer pools migrated between the 100- and 150-bp bands of the dsDNA ladder (Fig 1). The binding affinities, as evidenced by measured OD (optical density), increased gradually from 0.178 to 1.080. No further improvement in the binding affinities was observed after 12 rounds of selection (Fig 2).

**Fig 3:**
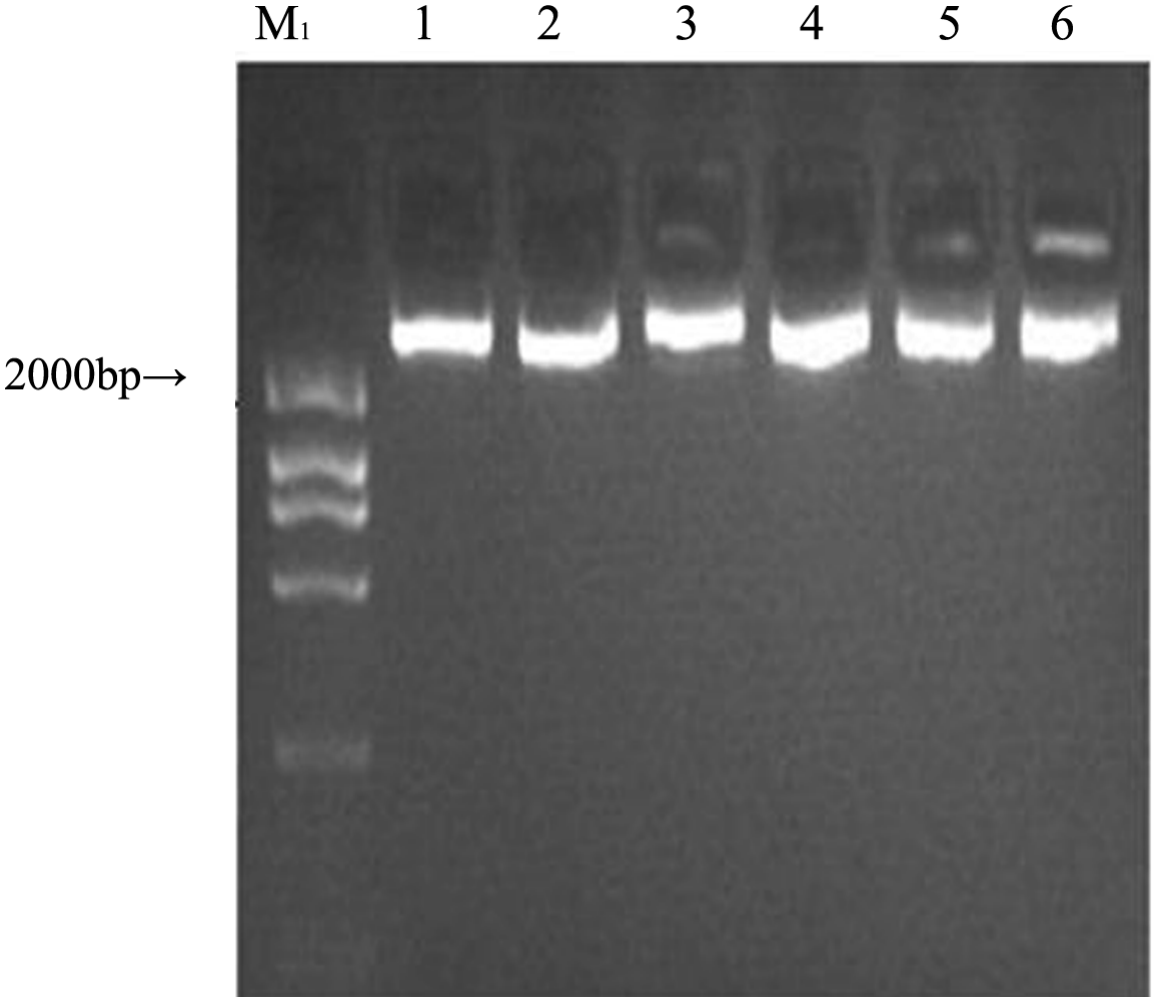
Agarose gel (0.8%) electrophoretic analysis. Lanes 1–6: the recombinant plasmid DNA from six positive clones. Lane M_1_: DNA ladder.

**Fig 4:**
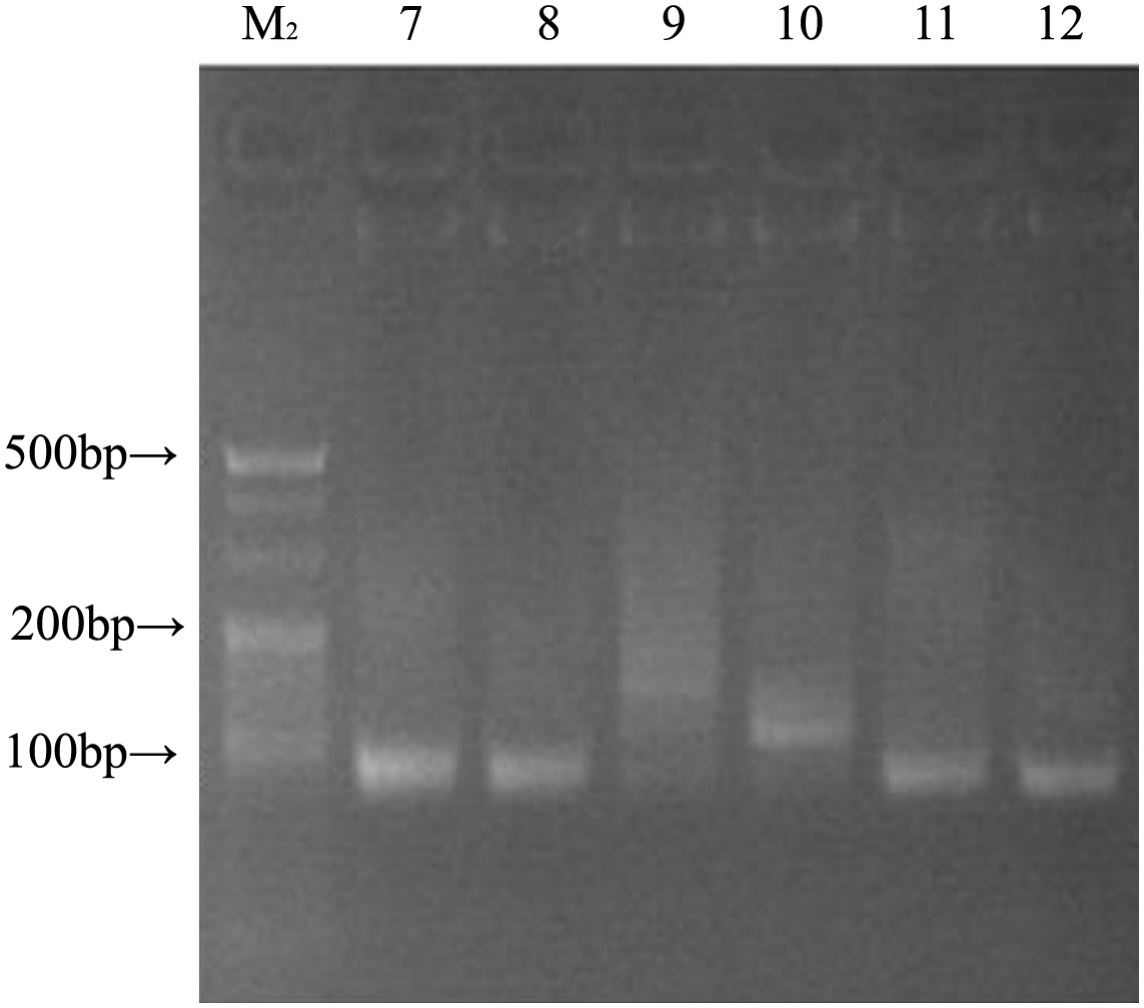
Agarose gel (3%) electrophoretic analysis. Lanes 7–12: PCR products for the insert fragments from the six recombinant plasmids; lane M_2_: DNA ladder.

An aptamer pool from the 12th round of selection was amplified. After electrophoresis, the recovered DNA was cloned into the pMD19-T vector and transformed into DH5α E. coli. After bacterial culture and IPTG (isopropyl β-D-thiogalactoside) screening, several recombinant plasmid DNAs from positive bacterial clones were extracted (Fig 3), and their insert fragments were amplified. Their PCR products migrated close to the 80-bp band of the dsDNA ladder on a 3% agarose gel, which matched the designed length (Fig 4). The recombinants were purified and analysed by sequencing. Seven aptamers were obtained, and their sequences are shown in Table 1. The sequence of Aptamer Y1 was the same as Aptamer Y14. DNAMAN software was used to analyse the secondary structures of the sequences. These seven aptamers represented the stem-loop or pocket structures with the appropriate structural characteristics for binding to the target.

**Table 1:** Sequence families after 12 rounds of SELEX.

Designation	sequence
AptamerY1	CCAGTAGCCATACCGGTTTGTGGATGGGGTGTATGCGAGTGATGGTGGATTG
AptamerY2	GAGCTTGTCGCAAAGGGTTCACCACATT
AptamerY6	CGATGGCCTGCCCGATTTTCCGGAGGGGCGATCTGCACATGCCGCAACGGG
AptamerY8	TGCGAGCATCTCGCTATCAGAGTGAGGTGATTTGTGCATA
AptamerY11	CGATGGCCTGCCCGATTTTCCGGAGGGGCGATCTGCACATGCCGCAGCGGG
AptamerY14	CCAGTAGCCATACCGGTTTGTGGATGGGGTGTATGCGAGT
AptamerY22	GATGATGGATTGGAGCTTGTCGCAAAGGGTTCACCACATT

**Fig 5:**
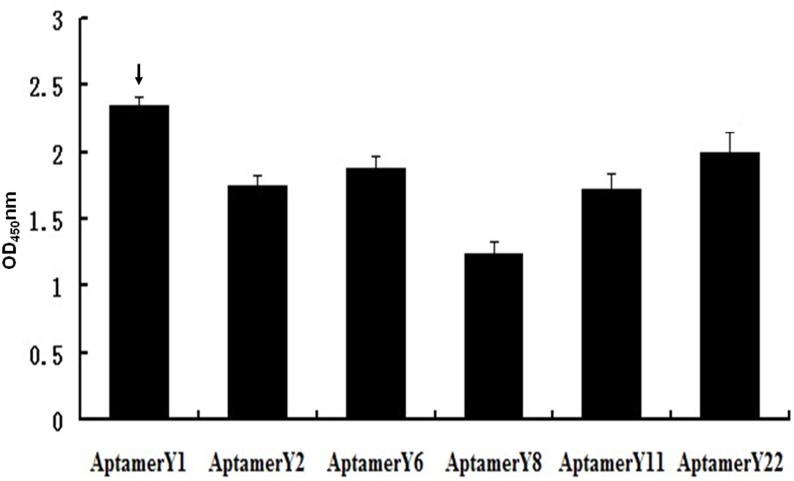
Relative binding affinities of the individual aptamers from the 12th pool as determined by ELONA analysis. All data shown were calculated as the mean ± SEM, and data were obtained from three independent experiments. The highest relative aptamer affinities are indicated by arrows.

Six biotin-labelled individual aptamers, with the exception of Aptamer Y14, were synthesized. The binding affinities between the individual aptamers and Orai1 were measured by ELONA. The OD values ranged between 1.18 and 2.31 (Fig 5). Aptamer Y1 had the highest affinity with an OD value of 2.31. Aptamer Y8 exhibited the lowest affinity. High-affinity Aptamer Y1 was selected for the next set of functional experiments in mast cells.

#### Effects of aptamers on human mast cell degranulation induced by IgE-crosslinking

**Fig 6:**
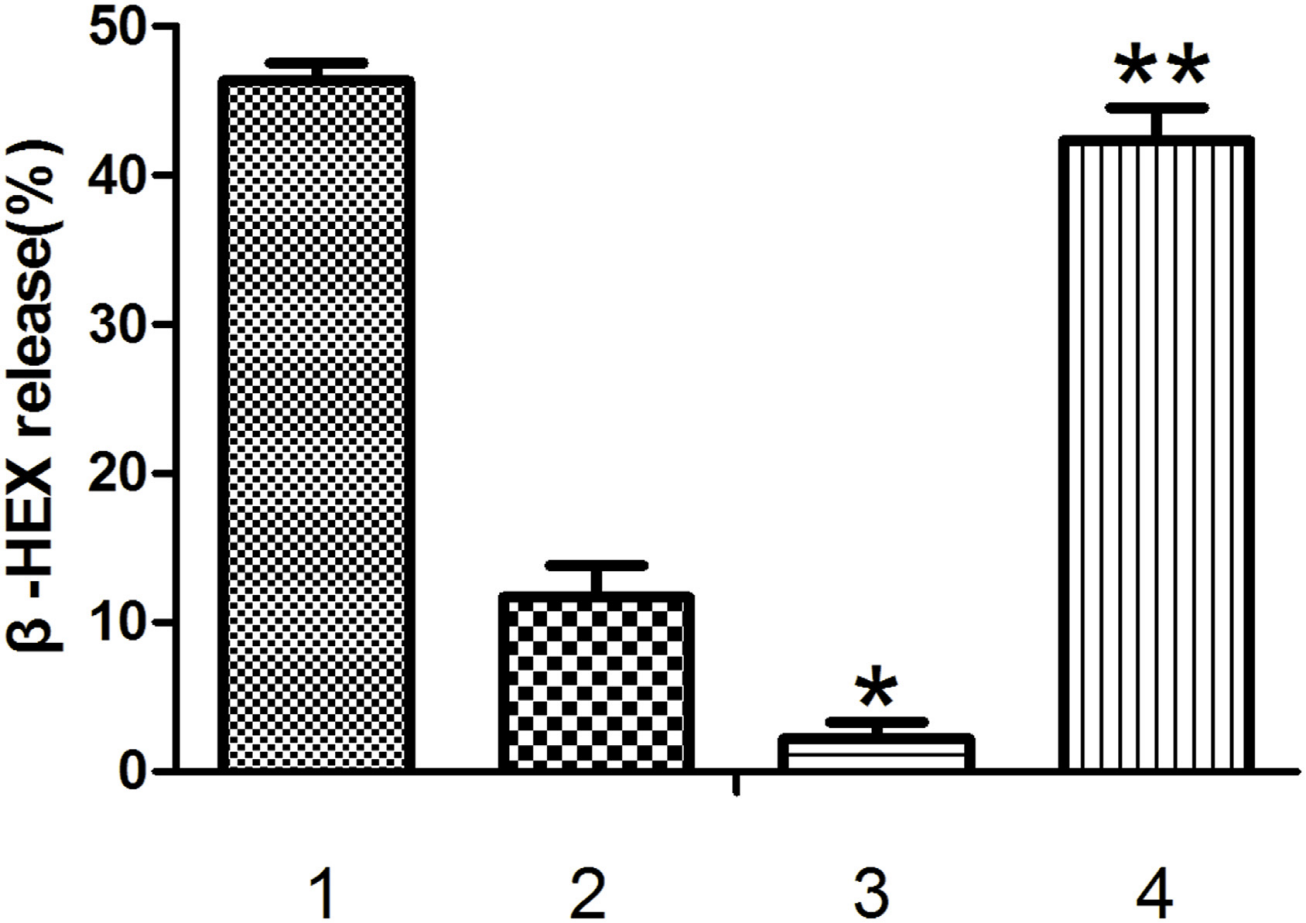
Effects of the aptamers on human mast cell degranulation induced by IgE-crosslinking. LAD2 were sensitized with 500 ng/mL biotinylated human IgE overnight. Cells were washed and resuspended (2×10^5^ cells/200 μL) in HEPES-Tyrode’s buffer and stimulated with 500 ng/mL streptavidin in the presence or absence of the indicated aptamers (final concentration of 2 μg/mL for 30 min. The cells were centrifuged, and the percent release of β-hexosaminidase (β-HEX) into the supernatant was calculated. β-HEX release (%) is expressed as the mean ± SEM for 3 separate experiments with LAD2 cells. * indicates p<0.05 compared with Group 2, and ** indicates *P* > 0.05 compared with Group 1 (0 nM aptamers) as determined by one-way ANOVA followed by Tukey's post-test. 1. No aptamers; 2. round one products of SELEX; 3. Aptamer Y1; 4. the control oligonucleotide ONT1.

β-hexosaminidase release is a reliable marker for mast cell degranulation. LAD2 cells were sensitized with biotin-IgE in the presence or absence of aptamers and then challenged with streptavidin to stimulate degranulation. Fig 6 illustrates that the release of the degranulation product β-hexosaminidase was inhibited by Aptamer Y1 at a final concentration of 2 μg/mL in LAD2 cells. At this concentration, Aptamer Y1 inhibited β-hexosaminidase release more than the products of the first round of SELEX (*P* < 0.05). By contrast, the control oligonucleotide ONT1 did not demonstrate any inhibition of degranulation (*P* > 0.05) as determined by β-hexosaminidase release from mast cells.

#### Dissociation constant (Kd) of Aptamer Y1

**Fig 7:**
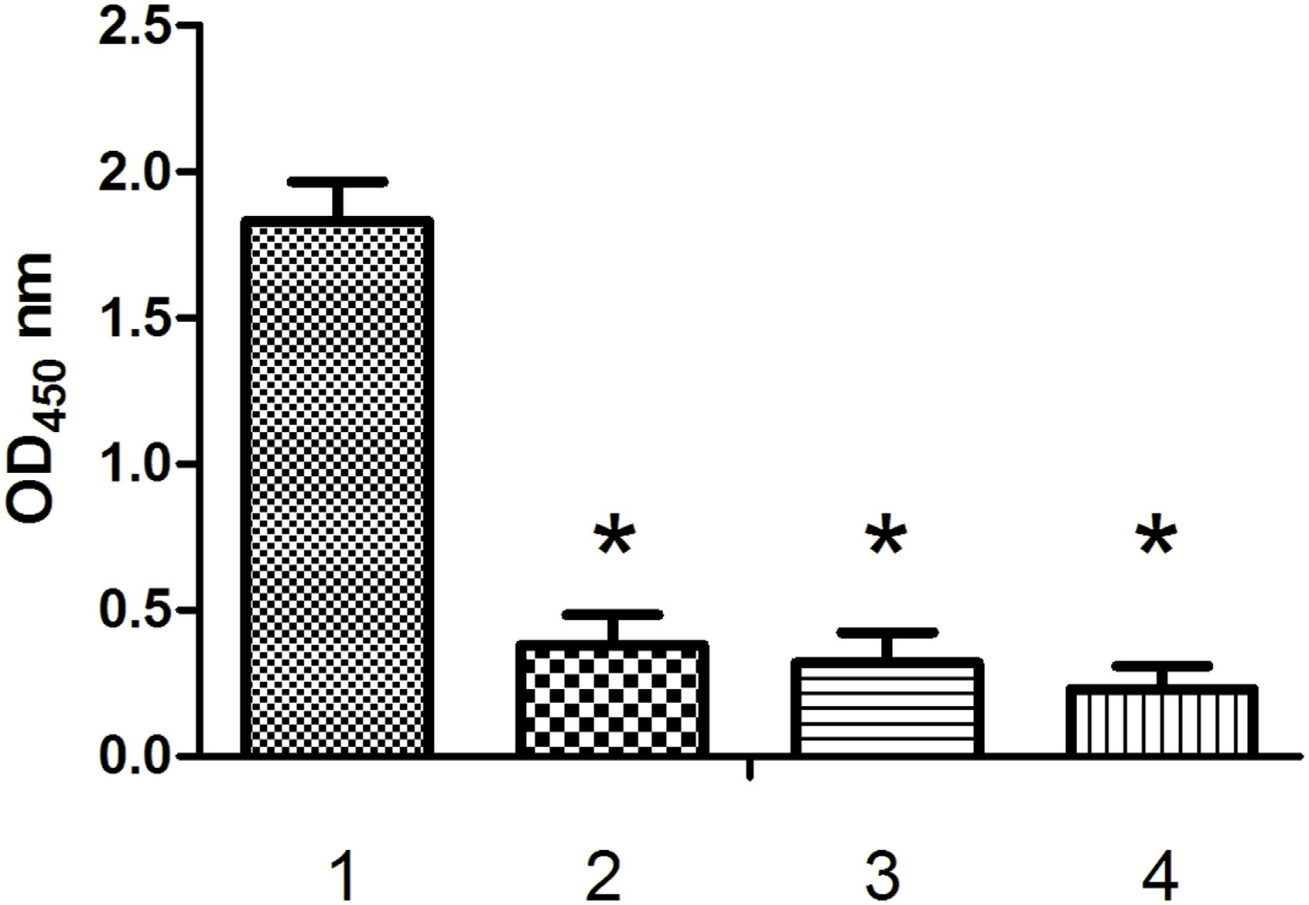
The binding affinities between Aptamer Y1 and different peptides. ELISA plates were coated with different peptides (1. the Orai1 peptide; 2. Cd1215; 3. IgEop; 4. BSA). Biotin-Aptamer Y1 was added into the wells. The absorbance was determined at 450 nm. All data shown were calculated as the mean ± SEM, and data were obtained from three independent experiments. * indicates *P* < 0.05 compared with the Orai1 peptide group.

To determine the binding affinity Kd values of Aptamer Y1, various concentrations of biotin-labelled Aptamer Y1 were added to the Orai1 peptide-coated wells, and the binding affinities were determined by ELONA. GraphPad Prism software was used to perform nonlinear curve fitting analysis for Kd calculation. The Kd values were determined to be 1.72 × 10^−8^ mol/L for Aptamer Y1 (Fig 7).

#### Specific binding assay

**Fig 8:**
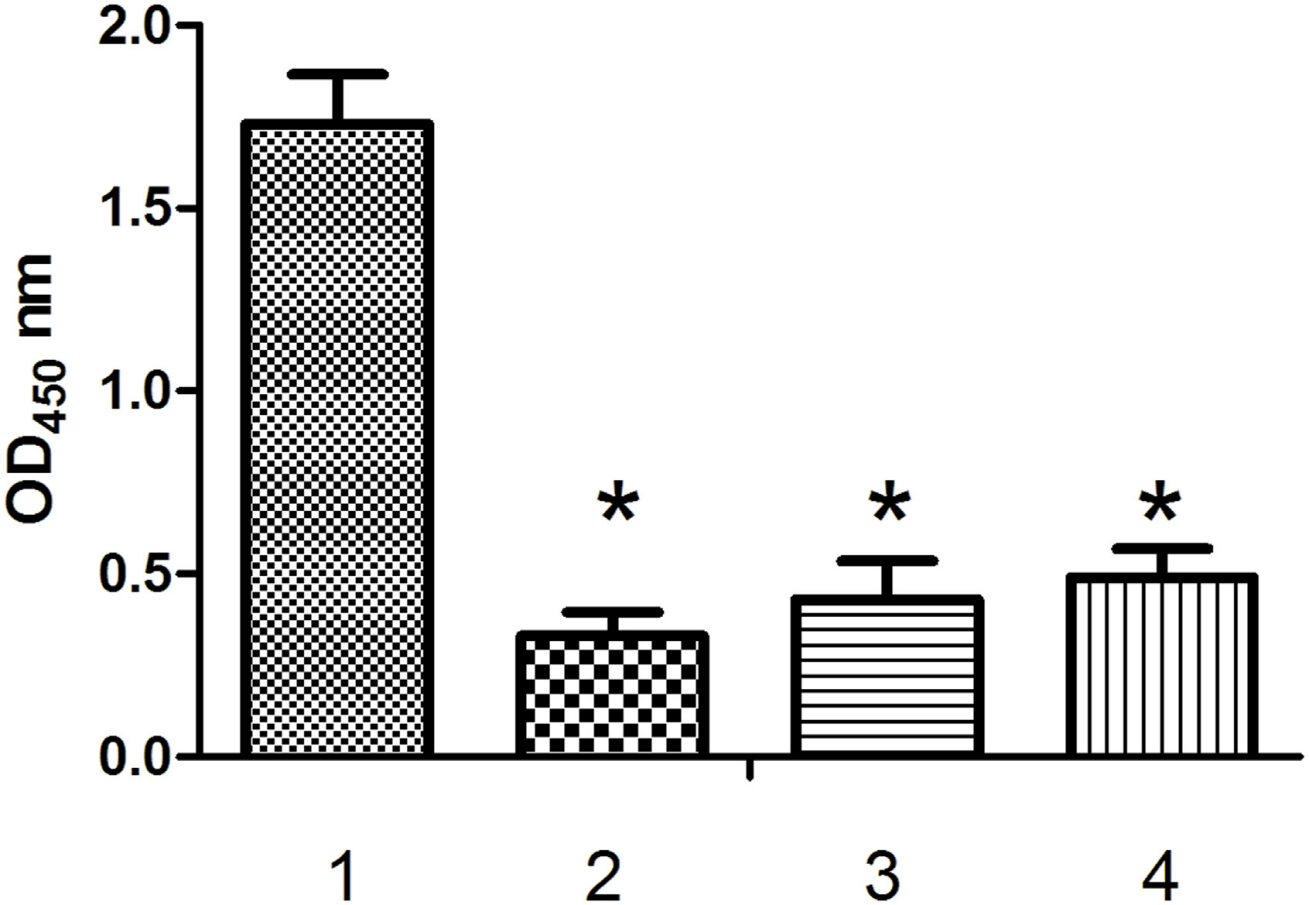
The binding affinities between the Orai1 peptide and different oligonucleotides. ELISA plates were coated with the Orai1 peptide. Different biotin-oligonucleotides were added into the wells (1. Aptamer Y1; 2. ONT1; 3. ONT2; 4. ONT3). The absorbance was determined at 450 nm. All data shown were calculated as the mean ± SEM, and data were obtained from three independent experiments. * indicates *P* < 0.05 compared with the Aptamer Y1 group.

**Fig 9:**
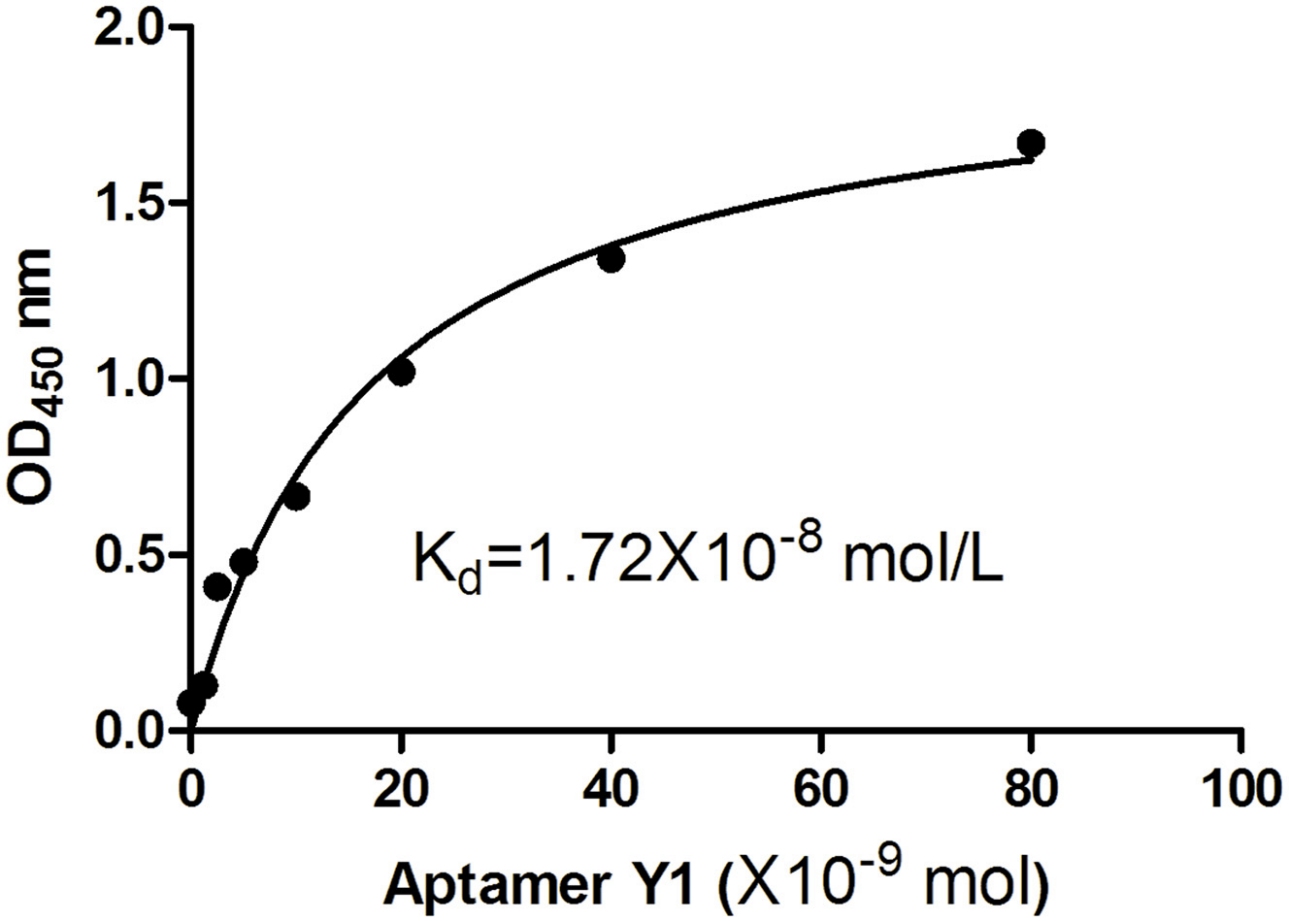
Measurement of the Kd values of Aptamer Y1 by ELONA. Binding between the Orai1 peptide and the biotin-labelled Aptamer Y1 was evaluated by measuring the absorbance at 450 nm. GraphPad Prism was used to perform nonlinear curve fitting analysis for the Kd calculation. All of the data are shown as the mean ± SEM, and data were from three independent experiments.

To test the specificity of the high-affinity interaction between Aptamer Y1 and the Orai1 peptide, ELONA experiments were performed with the peptides Cd1215, IgEop and BSA (Fig 8). The OD(450) value for the interaction between Aptamer Y1 and the Orai1 peptide was 1.86, whereas the OD(450) values for the interactions between Aptamer Y1 and Cd1215, IgEop, and BSA were 0.37, 0.29, and 0.18, respectively. Furthermore, we verified that biotinylated ONT1, ONT2 and ONT3 did not significantly bind to the Orai1 peptide compared with Aptamer Y1 (Fig 9). These results demonstrate that Aptamer Y1 not only binds the Orai1 peptide specifically but also with high affinity, and the Orai1 peptide does not bind significantly to other random oligonucleotide molecules.

#### Aptamer Y1 attenuated Ca2+ entry through SOCE in activated LAD2 cells

**Fig 10:**
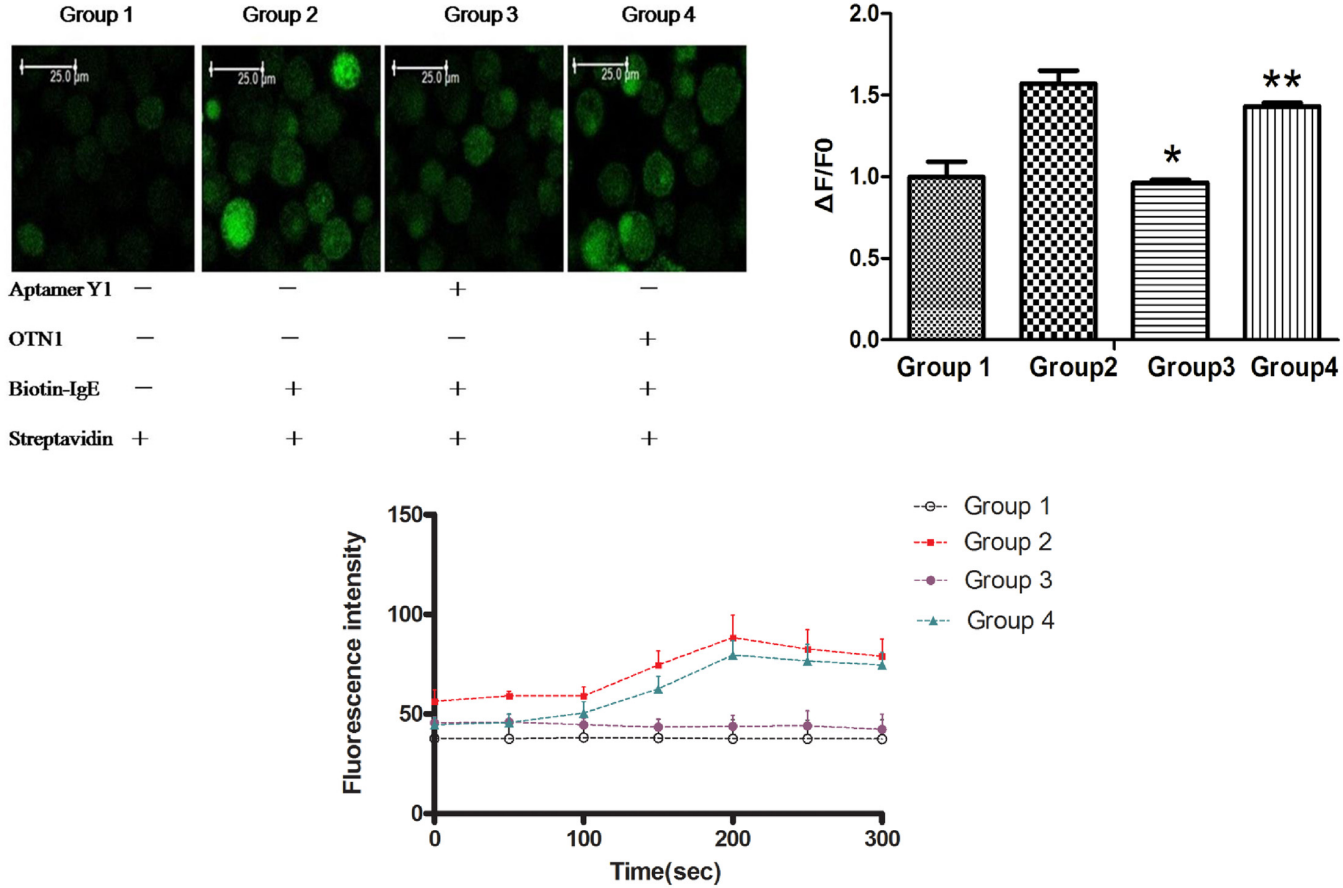
Aptamer Y1 reduced Ca2+ entry through SOCE in LAD2 cells. A 0.5-mL cell sample was prepared for each group at a concentration of 1×10^6^/mL. The cells were sensitized with biotin-IgE, and incubated for 2 h, followed by centrifugation at 1000 rpm × 5 min. Then, Tyrode's solution was added, and the cells were suspended and stained with 5 μmol/L Fluo-4-AM in the dark for 30 min, followed by centrifugation at 1000 rpm × 5 min. The cells were resuspended in Tyrode's solution and transferred into confocal dishes. Before observation, each sample received streptavidin (100 ng/mL) for activation. Experimental Group 1: No IgE group. In this group, the cells were not sensitized by IgE, and the other procedures were the same as those described above. Group 2: Biotin-IgE + streptavidin group. The cells were sensitized by biotin-IgE, and the other procedures were the same as described above. Group 3: Aptamer Y1 suppression group. After staining with Fluo-4-AM and resuspension, the cells were incubated with Aptamer Y1 (final concentration of 2 μg/mL) for 5 min. The proceeding steps were the same as those for Group 2. Group 4: ONT1 control group. After staining with Fluo-4-AM and resuspension, the cells were incubated with the oligonucleotide ONT1 (final concentration of 2 μg/mL) for 5 min. The proceeding steps were the same as those for Group 2. Observation of the cells by microscopy was performed immediately after activation with 500 ng/mL streptavidin. The data shown were calculated as the mean ±SEM, and data were from three independent experiments. (A) Fluorescent confocal images of LAD2 mast cells labelled with the Ca2+ indicator dye Fluo-4-AM. (B) Time-dependent antigen-stimulated increases in cytoplasmic Ca2+ detected by Fluo-4-AM fluorescence. (C) Aptamer Y1 reduced the value (ΔF/F0) of Group 2 significantly. *P < 0.05 compared with Group 2, *P* > 0.05 compared with Group 2.

Mast cell activation is dependent on an increase in cytosolic Ca^2+^ concentration, which is associated with release from intracellular stores and influx of external Ca^2+^ through SOCE. The effect of Aptamer Y1 on intracellular calcium mobilization in LAD2 cells was studied by monitoring intracellular Ca^2+^ fluorescence intensity. When the cells were sensitized with biotin-IgE, streptavidin dramatically induced intracellular Ca^2+^ elevation. The fluorescence intensity of the degranulation model group reached a peak (88.3 ± 11.2 vs. Ctrl 43.8 ± 5.5) 150 s after the addition of streptavidin, whereas the fluorescence intensity exhibited no significant changes 150 s after the addition of streptavidin in the Aptamer Y1 suppression group (*P* > 0.05). The fluorescence intensity of the OTN1 group reached its highest levels (79.5 ± 7.4) 150 s after streptavidin was added. The value (ΔF/F0) was significantly reduced by treatment with 2 μg/mL Aptamer Y1 in LAD2 cells (Fig 10). This result indicated that Aptamer Y1 had a direct suppressive effect on SOCE activity, and another oligonucleotide, OTN1, had no this effect. This experiment demonstrated the specific inhibitory effects of Aptamer Y1 on calcium influx and the specific binding between Aptamer Y1 and Orai1 molecules on the mast cell surface.

## Discussion

Store-operated Ca^2+^ entry (SOCE) is an important Ca^2+^ influx pathway in many resting cells, such as mast cells and T lymphocytes, that is regulated by the filling state of ER intracellular Ca^2+^ stores [15,16]. Emptying of ER Ca^2+^ results in activation of plasma membrane Ca^2+^ channels that mediate sustained Ca^2+^ influx, which is required for mast cell activation as well as refilling of Ca^2+^ stores [17,18]. The CRAC channel is the best characterized SOCE channel with well-defined molecular properties that modulate mast cell degranulation reactions, thereby playing an important role in mast cell-mediated diseases including allergies and asthma [19,20].

Recently, several human diseases have been linked to abnormal CRAC channel activities. A variety of small molecules able to block the CRAC channel have been identified, such as an imidazole derivative (SKF96365), 2-aminoethyldiphenyl borate (2-APB), a pyrazole derivative (YM58483), and Synta 66 [21–24]. SKF96365 can inhibit IL-2 production and activation in T lymphocytes with an IC (Inhibitory concentration) 50 of approximately 4 μmol/L [21]. YM-58483 potently inhibits store-operated sustained Ca^2+^ influx [23]. The compound Synta 66 was confirmed to occupy a central role in CRAC channels for mediator release from primary human lymphocytes and in airway inflammation and asthma symptoms in a preclinical model system [24]. These compounds are not specific because they operate in a variety of other transport processes. For example, SKF96365 blocked the transient receptor potential channel (TRP) C and TRP M channels with a similar potency as the CRAC channels. Therefore, molecules that specifically block Orai1 may be the focus and priority for CRAC channel research.

In this report, we describe the development of aptamers targeting an Orai1 peptide. ssDNA aptamers that bind to the Orai1 peptide with high affinity and high specificity were screened and identified from a random oligonucleotide library by SELEX. Through twelve rounds of SELEX, six aptamers with different sequences were identified. After determining the Kd values and estimating specificities, Aptamer Y1 with a Kd value of 1.72×10^−8^ mol/L was chosen as the ideal aptamer for mast cell tests. In the study, Aptamer Y1 inhibited the release of β-hexosaminidase and the intracellular calcium influx of LAD2 cells as determined in mast cell activation tests and confocal microscopy analysis. These types of effects exerted by Aptamer Y1 may result from the electric charges of its nucleotide. Aptamer Y1 binding with Orai1 may neutralize the negative charges in the first extracellular loop of Orai1 and disturb its molecular structure such that Orai1 molecules shut the gate in CRAC channels. To our knowledge, Aptamer Y1 is the first reported ssDNA aptamer specific to Orai1 and thus to CRAC channels. With its specificity and sensitivity, this aptamer may hold great promise for application in CRAC-related disorders.

Aptamers are single-stranded oligonucleotides with significant strategic properties in terms of their design, development and applications, to a greater extent than monoclonal antibodies [8]. One therapeutic aptamer specific to vascular endothelial growth factor, prescribed for age-related macular degenerative disease, has been approved by the FDA [25]. There are several aptamers currently in different phases of clinical trials. Almost all of these aptamers are specific to clinically important peptides or protein targets. Aptamers are promising artificial biological elements with broad ranges for application [26–28]. The aptamer developed in this study has not yet been studied *in vivo*, and mechanistic studies and biological evaluations are currently ongoing. The discovery of this novel Orai1-specific molecule represents a new strategy for the further design and development of CRAC channel inhibitors.

## References

[1] Di Capite JL, Bates GJ, Parekh AB. Mast cell CRAC channel as a novel therapeutic target in allergy. Curr Opin Allergy Clin Immunol. 2011;11:33–8. 10.1097/ACI.0b013e32834232b0 21150433

[2] Feske S, Wulff H, Skolnik EY. Ion channels in innate and adaptive immunity. Annu Rev Immunol. 2015;33:291–353. 10.1146/annurev-immunol-032414-112212 25861976 PMC4822408

[3] Muik M, Schindl R, Fahrner M, Romanin C. Ca^2+^ release-activated Ca2+ (CRAC) current, structure, and function. Cell Mol Life Sci. 2012; 69: 4163–76. 10.1007/s00018-012-1072-8 22802126 PMC3505497

[4] Prakriya M, Lewis RS. Store-operated calcium channels. Physiol Rev. 2015; 95: 1383–436. 10.1152/physrev.00020 26400989 PMC4600950

[5] Feske S, Gwack Y, Prakriya M, Srikanth S, Puppel SH, Tanasa B, et al A mutation in Orai1 causes immune deficiency by abrogating CRAC channel function. Nature. 2006;441: 179–85. 16582901 10.1038/nature04702

[6] Vig M, Peinelt C, Beck A, Koomoa DL, Rabah D, Koblan-Huberson M, et al CRACM1 is a plasma membrane protein essential for store-operated Ca^2+^ entry. Science. 2006;312:1220–3. 16645049 10.1126/science.1127883 PMC5685805

[7] Tuerk C, Gold L. Systematic evolution of ligands by exponential enrichment: RNA ligands to bacteriophage T4 DNA polymerase. Science. 1990;249:505–10. 2200121 10.1126/science.2200121

[8] Tabarzad M and Jafari M. Trends in the Design and Development of Specific Aptamers Against Peptides and Proteins Protein J. 2016;35:81–99. 10.1007/s10930-016-9653-2 26984473

[9] Yeromin AV, Zhang SL, Jiang W, Yu Y, Safrina O, Cahalan MD. Molecular identification of the CRAC channel by altered ion selectivity in a mutant of Orai. Nature. 2006;443:226–9. 16921385 10.1038/nature05108 PMC2756048

[10] Vig M, Beck A, Billingsley JM, Lis A, Parvez S, Peinelt C, et al CRACM1 multimers form the ion-selective pore of the CRAC channel. Curr Biol. 2006; 16:2073–9. 16978865 10.1016/j.cub.2006.08.085 PMC5685803

[11] Pan Q, Wang Q, Sun X, Xia X, Wu S, Luo F, et al Aptamer against mannose-capped lipoarabinomannan inhibits virulent Mycobacterium tuberculosis infection in mice and rhesus monkeys. Mol Ther. 2014;22:940–51. 10.1038/mt.2014.31 24572295 PMC4015227

[12] Tang XL, Zhou YX, Wu SM, Pan Q, Xia B, Zhang XL. CFP10 and ESAT6 aptamers as effective Mycobacterial antigen diagnostic reagents. J Infec. 2014;69: 569–80. 10.1016/j.jinf.2014.05.015 24968239

[13] Zhang B, Alysandratos KD, Angelidou A, Asadi S, Sismanopoulos N, Delivanis DA, et al Human mast cell degranulation and preformed TNF secretion require mitochondrial translocation to exocytosis sites: Relevance to atopic dermatitis. J Allergy Clin Immunol. 2011;127: 1522–3.e8. 10.1016/j.jaci.2011.02.005 21453958 PMC3381794

[14] Zhang B, Weng Z, Sismanopoulos N, Asadi S, Therianou A, Alysandratos KD, et al Mitochondria Distinguish Granule-Stored from de novo Synthesized Tumor Necrosis Factor Secretion in Human Mast Cells. Int Arch Allergy Immunol. 2012;159:23–32. 10.1159/000335178 22555146 PMC4278585

[15] Rysavy NM, Shimoda LM, Dixon AM, Speck M, Stokes AJ, Turner H, et al Beyond apoptosis: the mechanism and function of phosphatidylserine asymmetry in the membrane of activating mast cells. Bioarchitecture. 2014;4(4–5):127–37. 10.1080/19490992.2014.995516 25759911 PMC4914033

[16] Sutovska M, Adamkov M, Kocmalova M, Mesarosova L, Oravec M, Franova S. CRAC ion channels and airway defense reflexes in experimental allergic inflammation. Adv Exp Med Biol. 2013;756:39–48. 10.1007/978-94-007-4549-0_6 22836617

[17] Fahrner M, Derler I, Jardin I, Romanin C. The STIM1/Orai signaling machinery. Channels (Austin). 2013;7:330–43. 10.4161/chan.26742 24107921 PMC3913757

[18] Derler I, Schindl R, Fritsch R, Romanin C. Gating and permeation of Orai channels. Front Biosci (Landmark Ed). 2012;17:1304–22. 22201805 10.2741/3988

[19] Rice LV, Bax HJ, Russell LJ, Barrett VJ, Walton SE, Deakin AM, et al Characterization of selective calcium-release activated calcium channel blockers in mast cells and T-cells from human, rat, mouse and guinea-pig preparations. Eur J Pharmacol. 2013;704:49–57. 10.1016/j.ejphar.2013.02.022 23454522

[20] Di Capite JL, Bates GJ, Parekh AB. Mast cell CRAC channel as a novel therapeutic target in allergy. Curr Opin Allergy Clin Immunol. 2011;11:33–8. 10.1097/ACI.0b013e32834232b0 21150433

[21] Chung SC, McDonald TV, Gardner P. Inhibition by SK&F 96365 of Ca^2+^ current, IL-2 production and activation in T lymphocytes. Br J Pharmacol. 1994; 113: 861–8. 7858878 10.1111/j.1476-5381.1994.tb17072.x PMC1510420

[22] Prakriya M, Lewis RS. Potentiation and inhibition of Ca^2+^ release activated Ca^2+^ channels by 2-aminoethyldiphenyl borate (2-APB) occurs independently of IP3 receptors. J Physiol. 2001; 536: 3–19. 11579153 10.1111/j.1469-7793.2001.t01-1-00003.x PMC2278849

[23] Ishikawa J, Ohga K, Yoshino T, Takezawa R, Ichikawa A, Kubota H, et al A pyrazole derivative, YM-58483, potently inhibits store-operated sustained Ca^2+^ influx and IL-2 production in T lymphocytes. J Immunol. 2003; 170: 4441–9. 12707319 10.4049/jimmunol.170.9.4441

[24] Di Sabatino A, Rovedatti L, Kaur R, Spencer JP, Brown JT, Morisset VD, et al Targeting gut T cell Ca^2+^ release-activated Ca^2+^ channels inhibits T cell cytokine production and T-box transcription factor T-bet in inflammatory bowel disease. J Immunol. 2009;183:3454–62. 10.4049/jimmunol.0802887 19648266

[25] Nonaka Y, Yoshida W, Abe K, Ferri S, Schulze H, Bachmann TT, et al Affinity improvement of a VEGF aptamer by in silico maturation for a sensitive VEGF-detection system. Anal Chem. 2013;85: 1132–7. 10.1021/ac303023d 23237717

[26] Zhu J, Shang J, Jia Y, Pei R, Stojanovic M, Lin Q. Spatially selective release of aptamer-captured cells by temperature mediation. IET Nanobiotechnol. 2014; 8:2–9. 24888185 10.1049/iet-nbt.2013.0028 PMC6268195

[27] Ni J, Cozzi PJ, Duan W, Shigdar S, Graham PH, John KH, et al Role of the EpCAM (CD326) in prostate cancer metastasis and progression. Cancer Metastasis Rev. 2012; 31(3–4):779–91. 10.1007/s10555-012-9389-1 22718399

[28] Ni X, Castanares M, Mukherjee A, Lupold SE. Nucleic acid aptamers: clinical applications and promising new horizons. Curr Med Chem. 2011;18:4206–14. 21838685 10.2174/092986711797189600 PMC3260938

